# Investigating the phylogenetic history of toxin tolerance in mushroom‐feeding *Drosophila*

**DOI:** 10.1002/ece3.10736

**Published:** 2023-12-13

**Authors:** Theresa Erlenbach, Lauren Haynes, Olivia Fish, Jordan Beveridge, Sarah‐Ashley Giambrone, Laura K. Reed, Kelly A. Dyer, Clare H. Scott Chialvo

**Affiliations:** ^1^ Department of Genetics University of Georgia Athens Georgia USA; ^2^ Department of Biological Sciences University of Alabama Tuscaloosa Alabama USA; ^3^ Department of Biology Appalachian State University Boone North Carolina USA

**Keywords:** *amanita*, ancestral state reconstruction, biochemical novelty, mycophagy, phylogenetics

## Abstract

Understanding how and when key novel adaptations evolved is a central goal of evolutionary biology. Within the *immigrans‐tripunctata* radiation of *Drosophila*, many mushroom‐feeding species are tolerant of host toxins, such as cyclopeptides, that are lethal to nearly all other eukaryotes. In this study, we used phylogenetic and functional approaches to investigate the evolution of cyclopeptide tolerance in the *immigrans‐tripunctata* radiation of *Drosophila*. First, we inferred the evolutionary relationships among 48 species in this radiation using 978 single copy orthologs. Our results resolved previous incongruities within species groups across the phylogeny. Second, we expanded on previous studies of toxin tolerance by assaying 16 of these species for tolerance to α‐amanitin and found that six of them could develop on diet with toxin. Finally, we asked how α‐amanitin tolerance might have evolved across the *immigrans‐tripunctata* radiation, and inferred that toxin tolerance was ancestral in mushroom‐feeding *Drosophila* and subsequently lost multiple times. Our findings expand our understanding of toxin tolerance across the *immigrans‐tripunctata* radiation and emphasize the uniqueness of toxin tolerance in this adaptive radiation and the complexity of biochemical adaptations.

## Graphical abstract

Some species of *Drosophila* can develop in toxic mushrooms, which requires them to be tolerant of compounds such as α‐amanitin that are lethal to nearly all other eukaryotes. We infer the evolutionary history of α‐amanitin tolerance in the *immigrans‐tripunctata* radiation of *Drosophila*, where many species develop in mushrooms, and we find that α‐amanitin tolerance was likely present in the common ancestor of the radiation and has been lost several times. Our results highlight the complexity of biochemical adaptations and the uniqueness of this particular toxin tolerance.

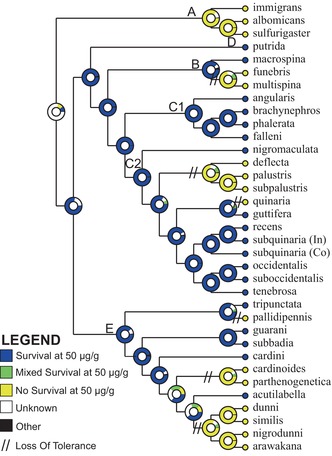

## 1 INTRODUCTION

Evolutionary novelties involve sets of genetic and phenotypic changes that can permit a species to enter a new niche, provide release from selective pressures like competition, or initiate adaptive radiations (Simpson, 1953, Heard & Hauser, 1995). Most research on adaptations has focused on the evolution of structures, for instance novel body parts (Broeckhoven et al., 2016; Hu et al., 2019; Ohde et al., 2018). Less studied are biochemical adaptations, though these are likely as important for the processes of adaptation and speciation in many organisms. Some key biochemical adaptations are venom production, chemical sequestration, and toxin tolerance (Giorgianni et al., 2020; Karageorgi et al., 2019; Reimche et al., 2022).

The genus *Drosophila* is comprised of ~2000 species, and it has been a model system for understanding the genetic basis of morphological changes and the selection pressures that underlie this variation (Markow, 2015, 2019; Markow & O'Grady, 2008). For instance, some well‐studied morphological traits that vary among closely related species include wing patterning, body pigmentation, body size, and sexual dimorphism (Chown & Gaston, 2010; Koshikawa, 2020; Markow & O'Grady, 2005, 2008; Massey & Wittkopp, 2016; Williams & Carroll, 2009). Among *Drosophila*, biochemical adaptations are more challenging to study but just as variable as morphological traits. In particular, there is tremendous variation among species in what host the larvae develop. The model species *Drosophila melanogaster* is known for consuming rotting fruit in orchards, but across the *Drosophila* genus, species use tree sap, slime flux, fruit, cactus, mushrooms, and vegetation as developmental hosts (Powell, 1997). Some *Drosophila* species are generalists and can develop on many different hosts, whereas other species are specialized on a single host species. A suite of behavioral and biochemical traits is associated with host use, where each trait may have a separate genetic basis; for instance, adults must locate and then oviposit on the host, and the larvae must be able to develop successfully in the host (Anholt, 2020; Bernays & Chapman, 2007; Etges, 2019; Markow, 2019; Whiteman & Pierce, 2008).

Among the most fascinating of the host adaptations are *Drosophila* that develop on toxic hosts, as these species have evolved the ability to tolerate compounds that kill most other species. Most species of *Drosophila* that are tolerant to host toxins are specialist feeders, which is thought to reduce competition and predation (Jones, 2005). For instance, the cactophilic *D. buzzatii* is associated with the non‐toxic prickly pear cactus (*Opuntia sulphurea*), whereas its sister species *D. koepferae* uses species within *Trichocereus* and *Cereus* genera that contain alkaloid secondary compounds (Hasson et al., 1992). Recent work has shown that enrichment of certain gene classes, such as responses to stress and metabolism of nitrogen compounds in columnar cacti dwellers like *D. keopferae*, may be responsible for the key adaptive differences between these species (Moreyra et al., 2023). Another example is *D. sechellia*, a specialist feeder on *Morina citrifolia* fruit (Louis & David, 1986), which contains octanoic acid, a fatty acid that is toxic to the closely related *D. simulans* (Furano et al., 2020; Jones, 2005; R'Kha et al., 1991).

Many *Drosophila* species utilize mushrooms as developmental hosts, and here we focus on the tolerance of certain *Drosophila* to cyclopeptides, which is the deadliest class of mushroom toxin (Bresinksy & Besl, 1990; Tang et al., 2016; Wieland, 1968). These toxins occur in some species of *Amanita* mushrooms, including the Death Cap (*A. phalloides*) and Destroying Angel (*A. virosa*), and are lethal to most eukaryotes, including humans. Fatalities are attributed to the amatoxin subclass, which act by binding to RNA polymerase II (RNAP II) and inhibiting mRNA production, leading to death (Lindell et al., 1970). Intriguingly, at least 12 *Drosophila* species in the *immigrans‐tripunctata* radiation use mushrooms containing cyclopeptide toxins as developmental hosts (Scott Chialvo & Werner, 2018). A species is defined as ‘tolerant’ if they can survive from egg to adult eclosion on a laboratory diet containing 50 μg/g α‐amanitin (Jaenike et al., 1983; Stump et al., 2011), though the toxic mushrooms these flies feed and develop on can contain up to 1600 μg of α‐amanitin per gram of dried mushroom (Wieland, 1968). Studies have shown that while flies survive without visible impacts to fitness on the mean α‐amanitin (250 μg/g) concentration, the extreme concentrations sometimes found in mushrooms (750–1000 μg/g) begin to affect the flies deleteriously (Jaenike, 1985).

The tolerance of mushroom‐feeding *Drosophila* to cyclopeptides is different from many other insect biochemical adaptations. First, typically only specialist feeders use hosts containing highly toxic compounds (Cornell & Hawkins, 2003; Whittaker & Feeny, 1971). Mushroom‐feeding *Drosophila*, however, are generalists that use a variety of fleshy non‐toxic and toxic fungi, and in some cases also use fruit and vegetation as developmental hosts (Jaenike & James, 1991; Lacy, 1984). Second, insects feeding on toxic hosts are often reported to be insensitive to the toxic compounds due to target site mutations (Holzinger & Wink, 1996; Karageorgi et al., 2019; Labeyrie & Dobler, 2004), but in toxin tolerant *Drosophila*, the target of amatoxins, RNAP II, does not contain mutations that prevent binding (Jaenike et al., 1983, Stump et al., 2011). It was found though that inhibition of Cytochrome P450s resulted in decreased tolerance in four of eight species assayed (Stump et al., 2011), suggesting P450s may be important to the mechanism of tolerance in some species. Third, the gut microbiome can contribute to detoxifying secondary metabolites encountered by some insects (Ceja‐Navarro et al., 2015; Shukla et al., 2018). In one species of α‐amanitin tolerant flies, however, alteration of the larval gut microbiome did not result in loss of tolerance (Griffin & Reed, 2020). Thus, these common mechanisms are not sufficient to explain toxin tolerance in mushroom‐feeding *Drosophila*.

Here, we study the evolution of cyclopeptide tolerance in mushroom‐feeding *Drosophila* using phylogenetic and functional approaches. A first step to understand the emergence of a novel trait is to infer its phylogenetic history, meaning when it evolved in the lineages where it is present. This inference requires a well‐resolved phylogeny. The evolutionary relationships in the *immigrans‐tripunctata* radiation, where cyclopeptide tolerance occurs, are poorly understood and vary depending on the species sampled and data used to construct the phylogeny (Dyer et al., 2011; Finet et al., 2021; Hatadani et al., 2009; Morales‐Hojas & Vieira, 2012; Perlman et al., 2003; Scott Chialvo et al., 2019; Spicer & Jaenike, 1996). Therefore, we first inferred the evolutionary relationships among an increased sampling of 48 species in the *immigrans‐tripunctata* radiation. We then expand upon previous studies that examined the effect of α‐amanitin (Jaenike, 1992; Jaenike et al., 1983; Stump et al., 2011) to characterize previously untested species' ability to survive on this toxin. We combine these empirical data with our species tree to construct a hypothesis of the ancestral state of toxin tolerance across this radiation. Our results suggest cyclopeptide tolerance arose once in the radiation and was then lost multiple times, and tolerance to α‐amanitin is widespread throughout the radiation.

## 2 METHODS

### 2.1 Fly stocks, taxon sampling, and RNA sequencing

We maintained fly stocks used in phylogenetic analyses and toxin tolerance assays at 22.5°C with 50% relative humidity and a 12:12 light: dark cycle. Flies were maintained on 4–24 Instant Drosophila Media (Carolina Biological) with the addition of a piece of commercial mushroom (*Agaricus bisporus*), necessary for fly mating and development in these species, and a dental cotton roll.

The 48 species used in the phylogenetic analyses are listed in Table 1. The sampling includes all four *testacea* group species and 18 of the 26 *quinaria* group species. There are two strains of *D. subquinaria*, one inland (Hinton, Alberta) and one coastal (Portland, Oregon), as these populations are known to be strongly isolated (Jaenike et al., 2006). Members from the *immigrans*, *tripunctata*, and *cardini* groups were also included. The two outgroup species were *D. grimshawi* and *D. virilis*. The *D. grimshawi*, *D. pruinosa*, *D. virilis*, and *D. quadrilineata* genomes were obtained from the 101 Drosophilid Genomes Project (Kim et al., 2021). The *D. innubila* genome assembly was downloaded from NCBI (GenBank accession GCA_004354385.2). For the remainder of the species (Table 1), we generated transcriptomes. Flies were collected 24 h after emergence, and RNA was extracted using the Omega Bio‐Tek EZNA Total RNA Kit 1 using an equal number of flash frozen males and females. Samples were sent to NovoGene for library prep and sequencing. Library preparation included poly‐A enrichment, and libraries were sequenced as 150 bp paired‐end reads.

**TABLE 1:** Species used in the study and their respective species groups, along with strain ID and collection location.

Species group (subgroup)	Species	Source	Collection location	Toxin tolerance to alpha‐amanitin	Feeding behavior	Assembly type	Number of reads	Complete and single copy	Complete and duplicated	Fragmented	Missing	Larvae per vial	Proportion survived at 0 μg/g	Proportion survived at 50 μg/g	𝛘^2^	*p*‐Value (df = 1)
*Bizonata*	*bizonata*	FK05‐12	Japanese stock center	NT	M^e^	T	21,916,714	72.6	24.9	1.5	1					
*Cardini (cardini)*	*cardini*	K. Dyer Lab	FL 2004 female 55	A^a^	M,F^f^	T	23,632,142	66.8	31	1.3	0.9					
	*cardinoides*	15,181–2191.06	Darien, Panama (1960).	B	NT	T	25,852,204	67.7	30.1	1	1.2	20	0.18	0.08	4.5	.0339
	*parthenogenetica*	15,181–2221.03	Nayarit, Mexico (2004). ISOFEMALE.	B	NT	T	21,672,259	69	27.9	1.9	1.2	15	0.417	0.0667	21.78	<.0001
	*polymorpha*	15,181–2231.01	Porto Alegre, Brazil.	NT	NT	T	25,606,327	66.1	32	1.2	0.7					
	*acutilabella*	K. Dyer Lab	Dark Eye, Obtained from J. Jaenike	A^a^	M,F^f^	T	20,312,496	67.1	29.3	2.1	1.5					
	*arawakana*	15,182–2261.03	Monkey Hill, St. Kitts, Caribbean Sea (2005). ISOFEMALE.	B	NT	T	24,433,756	71.3	26.1	1.5	1.1	25	0.328	0	63.95	<.0001
	*dunni*	15,182–2291.00	Mayaguez, Puerto Rico (1957).	B	NT	T	24,544,564	67.2	30.4	1.4	1	20	0.48	0	80.97	<.0001
	*nigrodunni*	15,182–2311.00	Turner Hall Woods, Barbados (1955).	B	NT	T	33,039,140	63.9	32.8	1.7	1.6	20	0.425	0.0125	48.08	<.0001
	*similis*	15,182–2321.00	St. George, Grenada (1957).	B	NT	T	30,029,959	64.8	32.4	1.6	1.2	25	0.267	0	29.829	<.0001
*Funebris*	*funebris*	15,120–1911.01	Mexico City, Mexico. ISOFEMALE.	B^a^	NT	T	26,617,202	61.8	35.7	1.1	1.4	15	0.517	0	53.017	<.0001
	*macrospina*	15,120–1931.08	Athens, Georgia (2004). ISOFEMALE.	A	NT	T	27,594,487	62.4	35.9	0.6	1.1	15	0.293	0.187	2.35	.125
	*multispina*	E‐24101	Sapporo, Japan (1958)	B	NT	T	28,547,997	61.6	36.1	1.3	1	15	0.433	0	20.696	<.0001
*Guarani*	*guarani*	E‐22301	Montero, Bolivia (1958)	A	NT	T	24,244,299	69	28.6	1.3	1.1	15	0.4	0.24	4.44	.351
	*subbadia*	15,172–2161.00	Veracruz, Mexico. ISOFEMALE	A	NT	T	21,780,171	71.5	25.6	1.6	1.3	15	0.827	0.627	7.68	.00558
*Immigrans*	*immigrans*	K. Dyer Lab	Edinburgh UK 2006	B^b^	F^g^	T	22,549,985	69.8	27.5	0.7	2					
	*quadrilineata*	*Kim* et al., *2021*, ^k^		NT	NT	G	NA	96.9	1.4	0.1	1.5					
*Immigrans (nasuta)*	*albomicans*	15,112–1751.00	Okinawa, Japan (1966).	B^a^	NT	T	21,490,220	70.1	24.4	3.8	1.7					
	*sulfurigaster*	15,112–1831.00	Queensland, Australia	B	NT	T	27,041,600	70.3	27.7	1	1	20	0.5	0	68.06	<.0001
*Incertae (sedis)*	*pruinosa*	*Kim* et al., *2021*, ^k^		NT	NT	G	NA	96.4	1.7	0.4	1.5					
*Macroptera*	*macroptera*	R. Unckless lab	Chirichahua mountains, AZ	NT	NT	T	23,917,190	74.4	23.4	1	1.2					
*Pallidipennis*	*pallidipennis*	15,210–2331.02	Caripe, Venezuela.	B	NT	T	29,104,675	65.6	31.1	2.2	1.1	15	0.267	0	23.669	<.0001
*Quinaria*	*angularis*	FK08‐14E1690	Japanese stock center	A^a^	M^a^	T	20,779,764	68.9	28.3	1.7	1.1					
	*brachynephros*	MT08‐1 E‐16501	Japanese stock center	A^a^	M^a^	T	19,081,239	72.1	25.2	1.8	0.9					
	*deflecta*	15,130–2018	Princeton, NJ (2000).	B^a^	V^g^	T	21,717,281	70.5	26.6	1.8	1.1					
	*falleni*	K. Dyer Lab	Athens GA	A^a,b,c^	M^g^	T	24,926,543	66	31.6	1.5	0.9					
	*guttifera*	15,130–1971.00	Species stock center	A^a,c^	M^g^	T	23,020,754	64.7	33.2	1.2	0.9					
	*innubila*	GCA_004354385.2	Genome sequence	NT	M	G	NA	96	2	0.5	1.5					
	*munda*	R. Unckless Lab	Chirichahua mountains, AZ	NT	M	T	19,362,169	71.9	25.7	1.6	0.8					
	*nigromaculata*	E‐14203	Hokkaido, Japan (2003)	A^a^	M,V,F^h^	T	28,541,917	64.9	33.3	0.9	0.9					
	*occidentalis*	K. Dyer Lab	Calgary AB	A	M	T	22,984,073	67.1	30.8	1.5	0.6	20	0.43	0.33	2.13	.144
	*palustris*	T. Werner Lab	Madison, WI	B^c^	V	T	26,952,050	64.2	33.8	1.2	0.8					
	*phalerata*	K. Dyer Lab	Munich Germany	A^a,c,d^	M^i^	T	25,948,513	68.2	29.8	0.9	1.1					
	*quinaria*	K. Dyer Lab	2004 Turkhill NY	B^a,c^	V^g^	T	28,613,871	66.9	31.1	1.5	0.5					
	*recens*	K. Dyer Lab	Peru, NY	A^a,b,c,d^	M^g^	T	28,843,862	61.8	36.3	1.1	0.8					
	*suboccidentalis*	K. Dyer Lab	Canmore, AB	A	M	T	27,295,501	71	26.7	0.8	1.5	20	0.24	0.34	2.43	.119
	*subpalustris*	15,130–2071.01	Species stock center	B^c^	V^g^	T	24,946,868	64.7	33.1	1.2	1					
	*subquinaria* Coastal	K. Dyer Lab	Mixed stock coast allo	A^a^	M^g^	T	29,785,320	67.1	30.8	1.4	0.7					
	*subquinaria* Inland	K. Dyer Lab	Calgary AB, 2016 ♀203	A^a^	M^g^	T	26,039,790	64.4	32.9	1.7	1					
	*tenebrosa*	K. Dyer Lab	Paradise AZ 2004	A	M	T	24,498,750	65.6	32.7	0.9	0.8	15	0.16	0.187	0.17	.68
	*transversa*	K. Dyer Lab	Uppsala Sweden	NT	M	T	29,482,063	64.4	33.2	1.1	1.3					
*Repleta*	*grimshawi*	*Kim* et al., *2021*, ^k^		NT	NT	G	NA	96.1	1.8	0.3	1.8					
*Testacea*	*neotestacea*	K. Dyer Lab	ST, Rochester NY 1990	NT	M^g^	T	23,921,807	62.1	36.1	0.9	0.9					
	*orientacea*	K. Dyer Lab	Kimura 2007	NT	M^h^	T	24,903,398	68.4	29.4	1.3	0.9					
	*putrida*	K. Dyer Lab	Rochester, NY 1991	A^b^	M^h^	T	26,638,271	68.5	29.2	1.4	0.9					
	*testacea*	K. Dyer Lab	Munich Germany	NT	M^j^	T	23,425,996	66.6	31.2	1.1	1.1					
*Tripunctata*	*tripunctata*	K. Dyer Lab	Athens, GA 2007 iso ♀1	A^a,b^	M,F^h^	T	31,632,471	64.7	33.1	1.3	0.9					
*Virilis*	*virilis*	*Kim* et al., *2021*, ^k^		NT	NT	G	NA	95.7	2.1	0.4	1.8					

*Note*: ⍺‐amanitin tolerance is defined as (a) survival at 50 μg/g ⍺‐amanitin or (b) no survival at 50 μg/g ⍺‐amanitin. Feeding behavior is described as M: mushrooms, V: decaying vegetation, and F: fermenting fruit. NT indicates ‘not tested’ as we do not have data for these. The type of assembly is listed as either transcriptome (T) or genome (G), with the number of reads included for species sequenced in this study. BUSCO v9 scores are based on the *metazoan* dataset. For species assayed for toxin tolerance in this study, we indicate the number of first‐instar larvae placed in each vial, the larvae to adult survival in control and toxin treatments, and the results of the statistical analysis. Citations are as follows: ^a^Stump et al. (2011), ^b^Jaenike et al. (1983), ^c^Spicer and Jaenike (1996), ^d^Jaenike (1985), ^e^Tuno et al. (2007), ^f^Colón‐Parrilla and Pérez‐Chiesa (1999), ^g^Werner and Jaenike (2017), ^h^Kimura et al. (1977), ^i^Werner and Jaenike (1995), ^j^Shorrocks (1977), and ^k^Kim et al. (2021).

### 2.2 Transcriptome assembly and dataset generation

Transcriptomes were assembled using Oyster River Protocol v2.2.2 (MacManes, 2018) with default parameters. Each transcriptome and genome was assessed for completeness using the *metazoan* BUSCO v3.0.2 dataset v9 (Simao et al., 2015). We used TOAST (Wcisel et al., 2020) to generate an individual fasta file of each BUSCO ortholog, using the functions ParseBuscoResults and ExtractBuscoSeqs in R v3.6.1 (R Core Team, 2019). For species with genomes (Table 1), we extracted the corresponding sequence for each ortholog from the genomes using samtools faidx v1.10 (Li et al., 2009). These sequences were appended to the ortholog fasta files generated from TOAST. The new fasta files for BUSCO orthologs were run through TOAST's MafftOrientAlign, MissingDataTable, and SuperAlign functions to generate an alignment file for each of the 978 individual orthologs and a concatenated alignment file containing all orthologs (506,575,152 bp).

### 2.3 Phylogenetic analyses

We generated species trees using maximum likelihood and coalescent‐based frameworks. To generate a maximum likelihood tree, we used the concatenated dataset generated above. We ran Model Finder (Kalyaanamoorthy et al., 2017) in IQ‐Tree v2.0.6 (Nguyen et al., 2015) to determine the model that best supported the data and generated a maximum likelihood species tree using that best‐fit model. We assessed branch support with 100 bootstrap replicates. To estimate the coalescent‐based tree, we first generated a gene tree for each ortholog using RAxML v8.1.12 (Stamatakis, 2014) with the GTRCAT model and 100 bootstraps. Bootstrapped gene trees were then used to estimate the species tree in ASTRAL‐III v5.6.1 (Zhang et al., 2018). All trees were visualized using Figtree v1.4.4 (http://tree.bio.ed.ac.uk/software/figtree/).

Since the loci used and site rate variation can influence a tree topology, we performed a sensitivity analysis (Buddenhagen et al., 2016; Dowdy et al., 2020). Briefly, sites in each ortholog were placed into 10 bins based on evolutionary rate using TIGER v2.0 (Cummins & McInerney, 2011). The first bin contained invariant sites, the last bin the fastest‐evolving sites, and the remaining sites were partitioned into the middle eight bins based on rate. The program AMAS (Borowiec, 2016) was used to concatenate the binned sequences for each ortholog. For each ortholog, we created eight alignments by sequentially adding more rapidly evolving bins (e.g., 2 + 3, 2 + 3 + 4, etc.); the first bin was excluded since it only included invariant sites. Using RAxML v8.1.12 (Stamatakis, 2014), maximum likelihood trees were generated for each alignment using the GTRCAT model and 100 bootstraps. We estimated the pairwise distance among trees using treeCMP v2.0.76 (Bogdanowicz et al., 2012). These trees were plotted using cmdscale in R v3.6.1 (R Core Team, 2019), and the Euclidean distance of each subsampled tree to their average center was calculated. We removed loci that did not have sites in all of the original site bins. The remaining 489 loci were then ranked based on this distance and placed equally into eight inclusion sets, from 61 to 489 loci, with each successive set containing more outlier trees. This was performed for each of the eight binning subsets to create a total of 64 locus‐inclusion sets. New alignments were generated for each locus‐inclusion set, and these were used to create a maximum likelihood tree for each locus in RAxML v8.1.12 (Stamatakis, 2014) with bootstrap support values. These bootstrapped trees were used in ASTRAL‐III v5.6.1 (Zhang et al., 2018) to identify support for the coalescent species tree for each locus‐inclusion set. To assess support for the maximum likelihood tree, we concatenated the alignment for each inclusion set, and then used the RAxML‐b flag to assess bootstrap support for each inclusion set. Heatmaps for each node of the tree were generated to show how support varies across these inclusion sets with the R package ggplot2.

### 2.4 Toxin tolerance assays

Previous studies of *Drosophila* cyclopeptide tolerance (Jaenike et al., 1983; Spicer & Jaenike, 1996; Stump et al., 2011) characterized tolerance to ⍺‐amanitin of 20 species distributed across six species groups in the *immigrans‐tripunctata* radiation. To conduct a more comprehensive examination of the evolution of ⍺‐amanitin, we assessed tolerance to ⍺‐amanitin in 16 additional species from six species groups (Table 1). Tolerance assays were conducted in 7.5 mL glass scintillation vials containing 250 mg of a mixture consisting of 73.5% Instant *Drosophila* food and 26.5% ground freeze dried *Agaricus bisporus* mushroom resuspended in either 1 mL of water or 50 μg/g ⍺‐amanitin (62.5 μg ⍺‐amanitin in 1 mL water), which was the concentration used previously to identify tolerant species (Jaenike et al., 1983; Stump et al., 2011). A 1.5 cm × 4 cm piece of cotton watercolor paper was added as a pupation substrate. Each replicate consisted of placing early first instar larvae (15–25 per species; Table 1) into the vial and observing survival to adult for 30 days, and we conducted five replicate vials of each treatment. We coded the survival of each individual larvae to adulthood using a binary strategy (0 = Dead; 1 = Survived). We quantified the impact of ⍺‐amanitin on survival for each species separately using a generalized linear model with a binomial distribution, logit link, and bias‐adjustment using the *brglm2* v0.8.2 package (Kosmidis, 2021). The only included model effect was toxin presence. Statistical analyses were completed in RStudio v2022.7.1.554.3 (RStudio Team, 2022).

### 2.5 Ancestral state reconstruction of toxin tolerance

To analyze how toxin tolerance has evolved along the *immigrans*‐*tripunctata* radiation, we inferred the ancestral state of toxin tolerance using RASP4 (Yu et al., 2020). This tool uses character state information for each species to infer the most likely ancestral state of each internal node of a phylogenetic tree. Data on toxin tolerance used for character state information is either from this study or previous studies (Jaenike et al., 1983, Spicer & Jaenike, 1996, Stump et al., 2011). These previous experiments were similar to ours. In Stump et al. (2011), feeding assays were performed in 4‐dram vials containing either no toxin or 50 μg/g ⍺‐amanitin, with first instar larvae added to the food. In Spicer and Jaenike (1996) and Jaenike (1985), tolerance was tested in 3‐dram glass vials along a range of ⍺‐amanitin concentrations, with eggs placed in the vials. In these studies, species that used toxic mushrooms as a developmental host in the wild survived on a concentration at or above 50 μg/g ⍺‐amanitin. Our categories were (A) at least 10% absolute survival on diet with 50 μg/g ⍺‐amanitin or (B) less than 10% absolute survival on diet with 50 μg/g ⍺‐amanitin. We used the Bayesian Binary MCMC (BBM) analysis in RASP with a fixed Jukes‐Cantor model and null root distribution for 5,000,000 generations using four chains sampled every 100 generations. The first 1000 samples were discarded as burn‐in. We used only species for which we had physiological data from ⍺‐amanitin and pruned the other species from the phylogeny, resulting in a phylogeny with 35 species. Ancestral state reconstruction was performed on both the coalescent and maximum likelihood phylogenetic trees.

## 3 RESULTS

### 3.1 Phylogenetic inference

To resolve the species tree for the *immigrans‐tripunctata* radiation, we generated transcriptomes or collected genomes for 48 species (Table 1). The final dataset contained 978 single‐copy orthologs from the BUSCO *metazoan* database. If a locus was duplicated, we used the highest scoring sequence from the BUSCO output. For our transcriptomes, the average number of single‐copy orthologs was 67.1%, with a duplicate average of 30.4%. The genomes had an average of 96.3% single‐copy orthologs and 1.8% average for duplicates. The percentage of all BUSCO categories (Complete and single‐copy, Complete and duplicated, Fragmented, and Missing) in each species are in Table 1.

**FIGURE 1:**
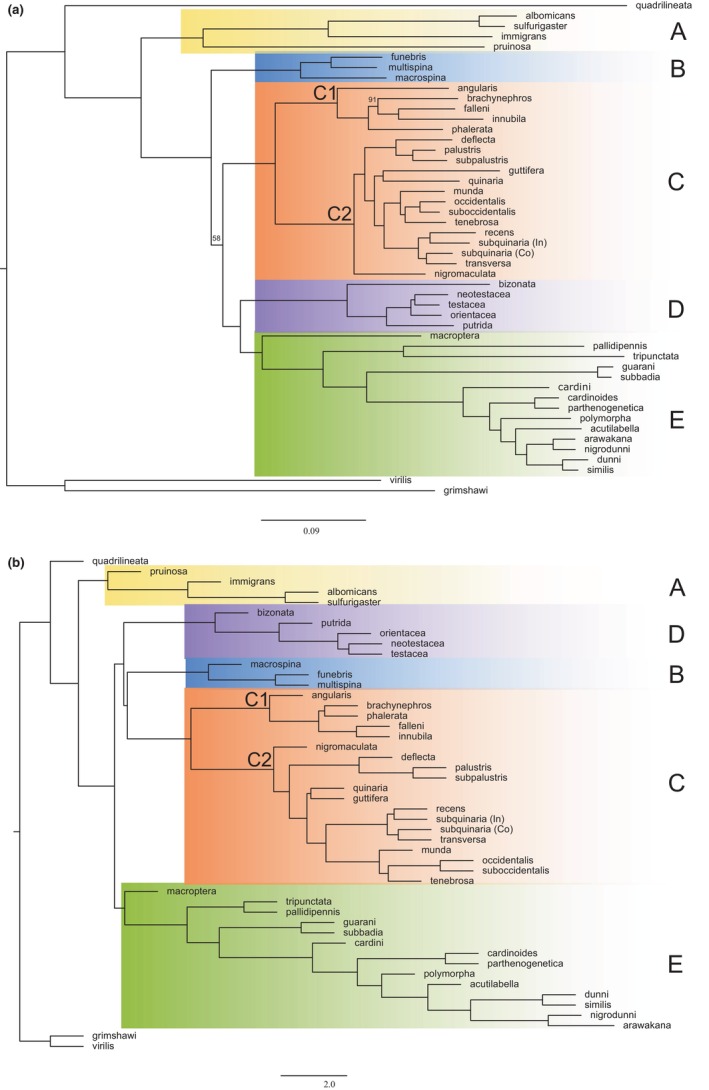
Species relationships for the *immigrans‐tripunctata* radiation generated by (a) maximum likelihood or (b) coalescent methods. Branch labels indicate bootstrap support and are only shown for branches with less than 100% support. Clade A in yellow represents the *immigrans* group and *D. pruinosa*, Clade B in blue is the *funebris* group, Clade C in orange is the *quinaria* group, with two subclades marked C1 and C2, Clade D in purple is the *testacea* group and *D. bizonata*, and Clade E in green is the *cardini* and *guarani* groups along with *D. tripunctata*, *D. macroptera*, and *D. pallidipennis*.

We used maximum likelihood and coalescent methods to generate a species tree from the 978 orthologs. The best model for our maximum likelihood tree was GTR + F + R10. Both the maximum likelihood topology (Figure 1a) and coalescent analysis (Figure 1b) produced five main well‐supported clades, which we refer to as clades A through E. Clade A consists of the *immigrans* group along with *D. pruinosa* and is sister to Clades B‐E. Clade B is the *funebris* group. Clade C is the *quinaria* group and contains two sister clades within it (C1 and C2). Clade D is the *testacea* group and *D. bizonata*. Clade E represents the *cardini* and *guarani* groups, along with *D. tripunctata*, *D. macroptera*, and *D. pallidipennis*. Within each clade, species relationships are identical between the two topologies other than the relationship of *D. brachynephros* and *D. phalerata* within Clade C1. Among deeper nodes, the relationships of Clades B, C, D, and E differ between the two phylogenies, with nearly all these basal nodes well supported in both trees.

**FIGURE 2:**
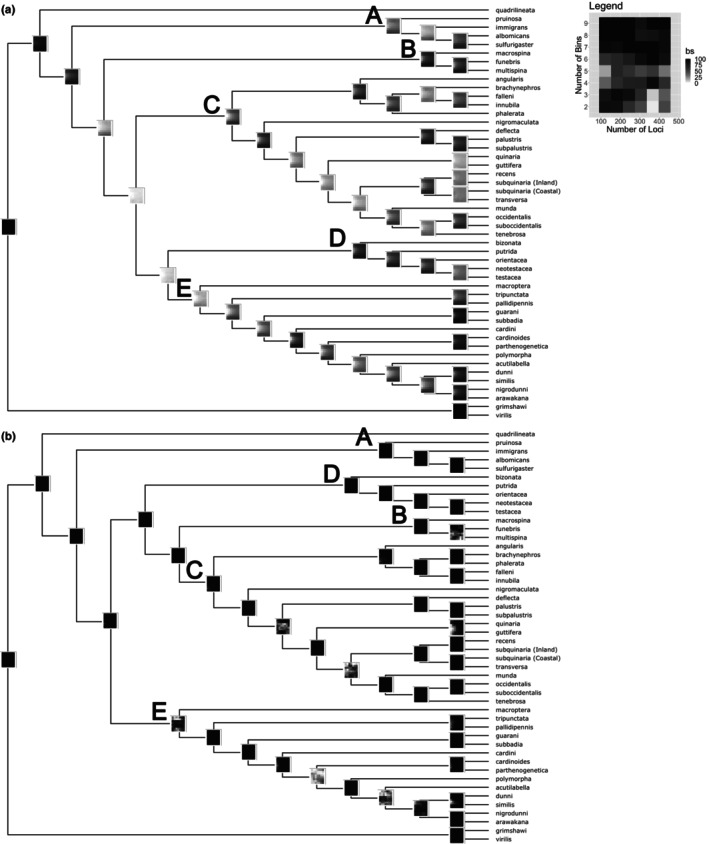
Sensitivity analysis for the (a) maximum likelihood species tree and (b) coalescent analysis species tree. Legend inset shows a randomly chosen heatmap, which gives the number of loci (X‐axis) and number of bins (Y‐axis) used. The color gradient indicates bootstrap support for the branches based on the loci and bin combinations in the sensitivity analysis, with white being 0 bootstrap support (bs) and black 100 bootstrap support.

We performed a sensitivity analysis to identify how the inclusion of faster‐evolving loci affects support of our phylogenies and determines the best species tree for further analyses. The sensitivity analysis found substantial variability for support of internal nodes in our maximum likelihood tree (Figure 2a), with most nodes having much lower bootstrap support, especially those leading to clades C and D + E. Specifically, we find lower bootstrap support for intermediate bin combinations (4–6) at all sets of loci for many nodes. This can most likely be attributed to a large portion of the sites in the locus‐inclusion set being largely conserved for bins 1–3, but the addition of more variable sites for intermediate bins (4–6) impacts tree reconstruction during bootstrapping. When most sites are conserved in lower bins (1–3), bootstrapping is going to produce similar topologies each time; however, when some of the sites in the alignment become variable as they are in intermediate bins, the resampling done during bootstrapping could produce topologies that vary from the main tree because there is random variation at a portion of the sites. Bootstrap support is recovered when we include more sites (bins 7–9), likely due to some of the variable sites being shared between closely related species and the resampling during bootstrapping occurring with a larger number of sites that are a more equal mix between conserved and variable.

In contrast, the coalescent phylogeny produced consistently higher bootstrap support values for most nodes independent of how many loci were included (Figure 2b). A few internal nodes had variability in bootstrap support, but the inclusion of more loci and variable sites increased their bootstrap support. This suggests that to resolve a species tree, fewer loci are necessary using a coalescent approach, whereas maximum likelihood approaches require larger loci sampling with site rate variation to obtain the same level of resolution. Thus, we concluded the coalescent topology is the best supported species tree.

### 3.2 Toxin tolerance

**FIGURE 3:**
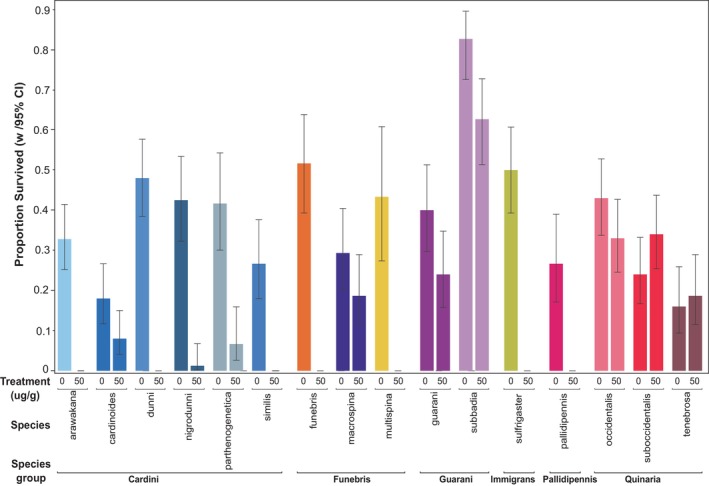
Proportion of first instar larvae that survived to adulthood on diets containing 0 or 50 μg/g ⍺‐amanitin. Species are clustered based on their species group and the error bars indicate the 95% binomial confidence intervals.

We assayed ⍺‐amanitin tolerance in 16 species, representing six species groups, that had not previously been tested. We reared larvae on diets with and without ⍺‐amanitin (50 μg/g) and measured the proportion that survived to adulthood (Figure 3, Table 1). In six species, at least 10% of larva survived to adulthood on the diet with toxin; these include *D. macrospina* in the *funebris* group, *D. guarani* and *D. subbadia* in the *guarani* group, and *D. occidentalis*, *D. suboccidentalis*, and *D. tenebrosa* in the *quinaria* group. Seven species produced no adults on ⍺‐amanitin treatment, which significantly reduced their survival (*p* < .0001). These include *D. arawakana*, *D. dunni*, and *D. similis* in the *cardini* group, *D. funebris* and *D. multispina* in the *funebris* group, *D. sulfurigaster* in the *immigrans* group, and *D. pallidipennis* in the *pallidipennis* group. The remaining three species in the *cardini* group (*D. cardinoides*, *D. nigrodunni*, *D. parthenogenetica*) had greater than zero survival on ⍺‐amanitin diet, but this was less than the 10% survival threshold to be defined as tolerant.

### 3.3 Evolution of toxin tolerance

**FIGURE 4:**
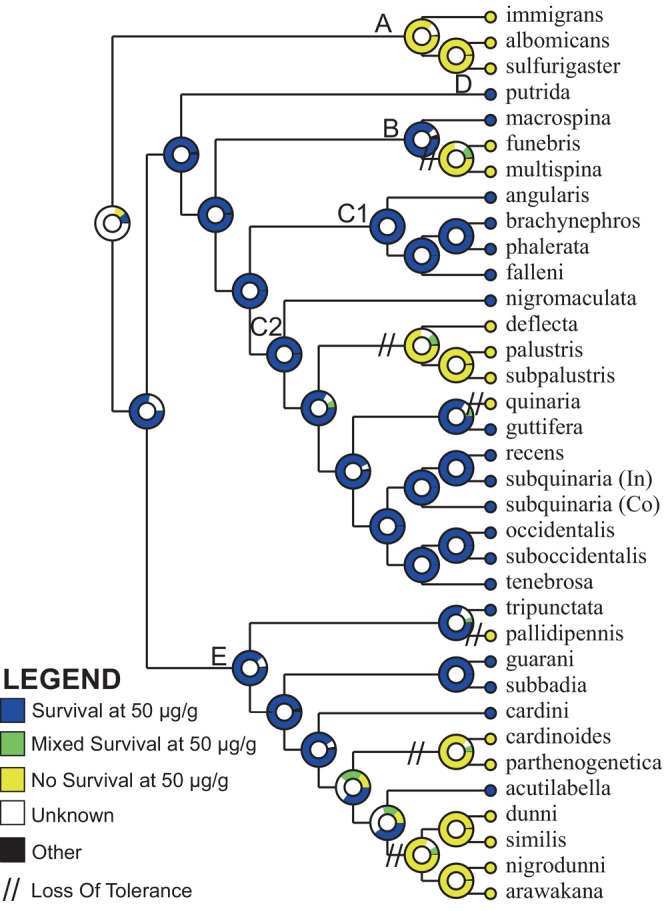
Ancestral state reconstruction of toxin tolerance evolution for the coalescent analysis species tree. The ancestral state of toxin tolerance is indicated on each branch by the circles, with the probability indicated by the colors, as shown in the legend. We also denote losses of tolerance along the tree.

With the knowledge that toxin tolerance varies within the *immigrans‐tripunctata* radiation, we sought to reconstruct the ancestral state of this trait. We examined toxin tolerance as a binary trait, using results from our toxin tolerance assays and previous studies (Jaenike et al., 1983; Spicer & Jaenike, 1996; Stump et al., 2011). We define tolerant as at least 10% survival at 50 μg/g of ⍺‐amanitin and susceptible as less than 10% survival at this concentration. There is uncertainty on toxin ancestry for our deepest node in the tree, but nodes in the phylogeny leading to Clades B‐E all indicate tolerance, which is subsequently lost at least five times (Figure 4). There is one loss of tolerance in the *funebris* group (Clade B) and two in the *quinaria* group (Clade C), including one in *D. quinaria* and another the common ancestor of *D. deflecta*, *D. palustris*, and *D. subpalustris*. Toxin tolerance is more variable in Clade E, where about half of the species assayed are susceptible. Tolerance was lost on the branch leading to *D. pallidipennis* and, for the *cardini* group, was either lost twice or lost once and then regained, and we suggest the former is most parsimonious.

## 4 DISCUSSION

Species within the *immigrans‐tripunctata* radiation of *Drosophila* exhibit a wide range of trait variation, including morphological characters, host preference, parasite prevalence, and toxin tolerance (Dombeck & Jaenike, 2004; Markow & O'Grady, 2008; Simunovic & Jaenike, 2006; Spicer & Jaenike, 1996; Stump et al., 2011; Werner et al., 2010). Of particular fascination is the tolerance found in many of these species to cyclopeptide toxins, as these are among the only eukaryotes known to consume and develop on these potent toxins (Scott Chialvo & Werner, 2018). Intriguingly, some species that are generalist feeders (i.e., on fleshy mushrooms, or a combination of mushrooms and rotting fruits and/or vegetative matter) are able to use cyclopeptide‐containing mushrooms as developmental hosts (Jaenike et al., 1983; Lacy, 1984), while some other more specialized mushroom feeders in the radiation (i.e., *D. funebris*) are not (Korneyev, 2010; Stump et al., 2011). These patterns run contrary to general ecological patterns of toxin‐tolerance being associated with host specificity. To broaden our understanding of cyclopeptide toxin tolerance and the evolutionary relationships within the *immigrans‐tripunctata* radiation, we generated a transcriptome phylogeny, conducted survival assays, and reconstructed toxin tolerance in the radiation.

When attempting to understand how traits among a lineage evolve, it is imperative to ensure that sufficient loci and a large enough taxon sampling are used in tree‐building. Recently, research on the superfamily Ephydroidea, which contains Drosophilidae, sought to tease apart inconsistencies with a broader sampling of species and increased number of nuclear genes (Winkler et al., 2022). This work reaffirmed and supported the need for broad taxonomic sampling across a lineage to best understand the evolution of species and the traits that encompass them. Phylogenetic studies of the *immigrans‐tripunctata* radiation have used either limited species sampling and/or few loci (Dyer et al., 2011; Hatadani et al., 2009; Izumitani et al., 2016; Scott Chialvo et al., 2019), resulting in incongruence among the recovered tree topologies. We reconstructed a species tree for 48 species in the *immigrans‐tripunctata* radiation using maximum likelihood and coalescent phylogenetic approaches with 978 single‐copy orthologs. Both trees produced the same five major clades, though there was variation in where they fell along the topology (Figure 1). Previous work using a smaller subset of this radiation could not confirm the monophyly of the *quinaria* group (Scott Chialvo et al., 2019). Our results support monophyly of the *quinaria* group, as the trees recovered from both analyses had the two subclades sister to one another (Figure 1). Interestingly, the *testacea* group (Clade D) has different locations along our topologies, either sister to Clade E (Figure 1a) or to the combined clades of B and C (Figure 1b). Due to these inconsistencies, we performed a sensitivity analysis and found the coalescent phylogeny produced the greatest support across all bin and loci combinations (Figure 2b). With factors such as incomplete lineage sorting giving high support to an incorrect topology generated with a concatenated loci dataset (Kubatko & Degnan, 2007; Mendes & Hahn, 2018; Roch & Steel, 2015), it is not surprising the coalescent topology was the most consistent. Other published phylogenies show similar topologies, including the *testacea* and *funebris* groups sharing immediate ancestry (Finet et al., 2021; Zhang et al., 2021).

We surveyed the ability of 16 species from six species groups to develop on diet containing α‐amanitin, six of which have a greater than 10% survival rate (Figure 3). Three are members of the *quinaria* species group, which is known to include many tolerant species (Jaenike et al., 1983; Spicer & Jaenike, 1996; Stump et al., 2011). The other three are from the *guarani* and *funebris* species groups. The *guarani* group is neotropical (Penafiel‐Vinueza & Rafael, 2018), and very little is known about their natural history. In the *funebris* group, one of the three species tested, *D. macrospina*, survived at a high level on the ⍺‐amanitin diet. This species breeds in woodlands near streams (Mainland, 1942; Miller et al., 2017), but nothing is known about its host usage. Another susceptible member of the group, *D. funebris*, is a specialized feeder on polypore, shelf fungi (Korneyev, 2010); this species did not produce any adults on the 50 μg/g α‐amanitin diet. This is consistent with earlier studies of toxin tolerance in *D. funebris* (Stump et al., 2011). Interestingly, we find variable tolerance in the *cardini* group. For example, there is weak survival (<10%) on 50 μg/g α‐amanitin diet for *D. cardinoides*, *D. parthenogenetica*, and *D. nigrodunni*. *Drosophila acutilabella*, another member of the *cardini* group, is known to feed on mushrooms and has higher survival on toxin food (21%) (Stump et al., 2011). Given that all previously tested α‐amanitin‐tolerant species use fleshy mushrooms as hosts (Jaenike et al., 1983; Lacy, 1984), our results suggest cyclopeptide tolerance is associated with this specific feeding behavior, and the presence of toxin tolerance in a species could serve as an indicator of its host usage.

Including our results, cyclopeptide tolerance has been identified in seven species groups in the *immigrans‐tripunctata* radiation based on whether larvae can survive on a diet containing 50 μg/g ⍺‐amanitin. While the 50 μg/g concentration is below the mean ⍺‐amanitin concentration (250 μg/g) in toxic *Amanita* mushrooms (Tyler Jr. et al., 1966; Yocum & Simons, 1977), only species that consume fleshy mushrooms can survive on this concentration (Jaenike et al., 1983; Stump et al., 2011). Our findings indicate that cyclopeptide tolerance is more broadly distributed within the *immigrans‐tripunctata* radiation than previously known, and our ancestral state reconstruction of toxin tolerance finds that α‐amanitin tolerance evolved early in the radiation and was lost multiple times (Figure 4). These results are nearly identical if we use the maximum‐likelihood tree for trait reconstruction (results not shown). This is consistent with Spicer and Jaenike (1996), who proposed that toxin tolerance evolved once and was lost multiple times in the *quinaria* group. We note that Stump et al. (2011) showed that inhibition of Cytochrome P450s affected toxin tolerant species differently but did support tolerance as the ancestral state.

Unlike other *Drosophila* that specialize on a single toxic host, most mushroom‐feeding *Drosophila* are generalists on fleshy basidiomycetes and only a small portion of their diet is expected to consist of toxic mushrooms. The selective forces that maintain cyclopeptide tolerance in mushroom‐feeding *Drosophila* remain unclear. One potential selective benefit is that toxin tolerance has been suggested to be associated with reduced parasitism (Jaenike, 1985). Specifically, the prevalence of nematode parasitism in flies collected from toxic mushrooms is significantly lower than in flies collected from non‐toxic mushrooms, suggesting that nematodes are not tolerant of toxins. Thus, ⍺‐amanitin‐containing mushrooms provide a source of respite from nematode parasitism and the subsequent infertility that results, increasing their fitness (Jaenike, 1985). However, a study in *D. putrida* found no direct effects of ⍺‐amanitin on the host–parasite interaction, suggesting that selection due to resource competition is a more likely mechanism to maintain tolerance to toxin (Debban & Dyer, 2013). Unlike many hosts, mushrooms are a patchy and ephemeral resource, and a newly emerged mushroom‐feeding fly must find a new mushroom host promptly because its developmental host has disintegrated. If hosts are limited, the ability to use both toxic and non‐toxic mushrooms may be strongly favored. This tolerance is likely costly; however, as in all known instances where a species has switched from a mushroom to a non‐mushroom host (i.e. *D. quinaria*), it has lost toxin tolerance.

Our study characterized toxin tolerance using a single purified toxin, ⍺‐amanitin, and one strain per *Drosophila* species; this did not capture any intraspecific variation in the trait. Other recent work examined four species in the *immigrans‐tripunctata* radiation and found both intra‐ and interspecific variation in tolerance to a natural mix of toxins found in *Amanita bisporus* mushrooms (Kokate et al., 2022). The Kokate et al. (2022) study included *D. neotestacea* (*testacea* group), which has not been tested for tolerance to ⍺‐amanitin specifically. With our expanded understanding of toxin tolerance across the *immigrans‐tripunctata* radiation, future studies should examine tolerance of more species to a natural mix of toxins, as toxic *Amanita* mushrooms can contain over 10 different cyclopeptide toxins. Further, examining tolerance in multiple strains per species will further characterize intraspecific variation in this trait, refine ancestral state reconstruction of toxin tolerance, and the selective pressures that maintain this important adaptation.

In conclusion, the combination of our phylogenetic and functional approaches allowed us to disentangle previous incongruities in the phylogeny, as well as expand our understanding on toxin tolerance to ⍺‐amanitin. Our results suggest that the evolution of toxin tolerance in mushroom‐feeding *Drosophila* is ancestral in the *immigrans‐tripunctata* radiation and was lost several times across the radiation. Future studies should expand from assaying tolerance with ⍺‐amanitin to the full mix of toxins found in *Amanita* mushrooms to explore factors that contribute to the evolution and maintenance of tolerance within and among *Drosophila* species.

## AUTHOR CONTRIBUTIONS

**Theresa Erlenbach:** Conceptualization (equal); data curation (equal); formal analysis (equal); investigation (equal); writing – original draft (equal); writing – review and editing (equal). **Lauren Haynes:** Investigation (supporting). **Olivia Fish:** Investigation (supporting). **Jordan Beveridge:** Investigation (supporting). **Sarah‐Ashley Giambrone:** Investigation (supporting). **Laura K. Reed:** Conceptualization (equal); funding acquisition (equal); project administration (equal); supervision (equal); writing – review and editing (equal). **Kelly A. Dyer:** Conceptualization (equal); funding acquisition (equal); project administration (equal); supervision (equal); writing – original draft (equal); writing – review and editing (equal). **Clare H. Scott Chialvo:** Conceptualization (equal); formal analysis (equal); funding acquisition (equal); project administration (equal); supervision (equal); writing – original draft (equal); writing – review and editing (equal).

## CONFLICT OF INTEREST STATEMENT

The authors have no conflicts of interest to declare.

## Data Availability

All code is available in the GitHub repository at https://github.com/trm76056/phylogenetic‐tree. Raw RNA sequencing fastq files are deposited in Dryad (DOI: 10.5061/dryad.hdr7sqvq2).
